# Amorphous Iridium Oxide-Integrated Anode Electrodes with Ultrahigh Material Utilization for Hydrogen Production at Industrial Current Densities

**DOI:** 10.1007/s40820-024-01411-7

**Published:** 2024-05-24

**Authors:** Lei Ding, Kui Li, Weitian Wang, Zhiqiang Xie, Shule Yu, Haoran Yu, David A. Cullen, Alex Keane, Kathy Ayers, Christopher B. Capuano, Fangyuan Liu, Pu-Xian Gao, Feng-Yuan Zhang

**Affiliations:** 1 Department of Mechanical, Aerospace and Biomedical Engineering, University of Tennessee, Knoxville, TN 37996 USA; 2 Oak Ridge National Lab, Center for Nanophase Materials Sciences, Oak Ridge, TN 37831 USA; 3 Nel Hydrogen, Wallingford, CT 06492 USA; 4 Institute of Materials Science, University of Connecticut, Storrs, CT 06269 USA; 5 Department of Materials Science and Engineering, University of Connecticut, Storrs, CT 06269 USA

**Keywords:** Ionomer-free, Amorphous IrO_*x*_ electrodes, Ultrahigh material utilization, Scalable electrodeposition, Hydrogen production

## Abstract

Herein, ionomer-free amorphous iridium oxide (IrO_*x*_) thin electrodes are first developed as highly active anodes for proton exchange membrane electrolyzer cells (PEMECs) via low-cost, environmentally friendly, and easily scalable electrodeposition at room temperature. Combined with a Nafion 117 membrane, the IrO_*x*_-integrated electrode with an ultralow loading of 0.075 mg cm^−2^ delivers a high cell efficiency of about 90%, achieving more than 96% catalyst savings and 42-fold higher catalyst utilization compared to commercial catalyst-coated membrane (2 mg cm^−2^). Additionally, the IrO_*x*_ electrode demonstrates superior performance, higher catalyst utilization and significantly simplified fabrication with easy scalability compared with the most previously reported anodes. Notably, the remarkable performance could be mainly due to the amorphous phase property, sufficient Ir^3+^ content, and rich surface hydroxide groups in catalysts. Overall, due to the high activity, high cell efficiency, an economical, greatly simplified and easily scalable fabrication process, and ultrahigh material utilization, the IrO_*x*_ electrode shows great potential to be applied in industry and accelerates the commercialization of PEMECs and renewable energy evolution.

**Supplementary Information:**

The online version contains supplementary material available at 10.1007/s40820-024-01411-7.

## Highlights

- Electrodeposited amorphous IrO_*x*_ thin electrodes are first developed for proton exchange membrane electrolyzer cells.
- An ultralow loading of 0.075 mg cm^−2^ archives superior performance to catalyst-coated membrane (2 mg cm^−2^).
- > 96% of catalyst savings and > 42-fold higher catalyst utilization are demonstrated.

**Supplementary Information:**

The online version contains supplementary material available at 10.1007/s40820-024-01411-7.

## Graphical abstract

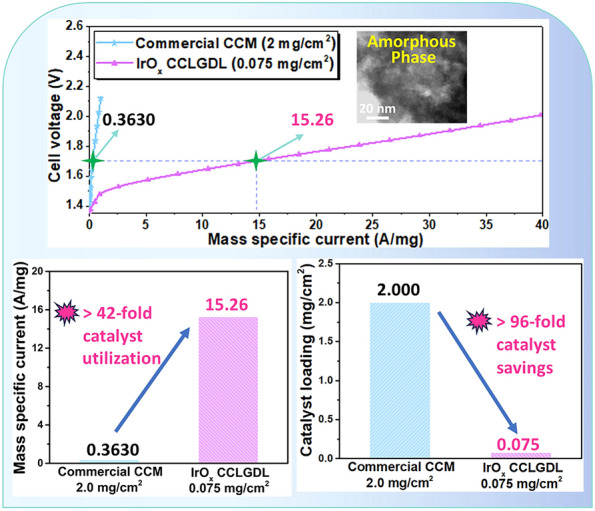

## Introduction

To solve the global energy crisis and environmental pollution resulting from the combustion of fossil fuels, developing highly efficient energy conversion and storage technologies is a promising strategy [1–6]. Water electrolyzers, one of the most ideal alternative energy storage and carrier materials to fossil fuels, are devices driven by electricity from regenerative and clean energy sources [7–12]. As the U.S. Department of Energy (DOE) issued the first Energy Earth short, Hydrogen Shot, to reduce the cost of clean hydrogen to $1 per 1 kg in 1 decade (“1 1 1”), ever more research resources and efforts have been devoted to the development of clean hydrogen. The large-scale application of water electrolyzers is impeded by high cost and the scarcity of platinum group metals (PGM), especially for the anode side, which involves sluggish reaction kinetics [13, 14]. Hence, developing highly efficient water electrolyzers at low cost is desired to accelerate the renewable energy revolution.

Proton exchange membrane electrolyzer cells (PEMECs) have attracted extensive attention due to their compact design, quick start-up, high efficiency, and low maintenance cost. In PEMECs, membrane electrode assembly (MEA) plays an important role. Conventional MEA configuration design is a catalyst-coated membrane (CCM) combined with liquid–gas diffusion layers (LGDLs), which normally requires high PGM loadings (up to 1–3 mg cm^−2^) to ensure cell performance and durability [15–17]. However, Mo and co-authors discovered that the ionomer-mixed catalyst layer in the CCM showed limited electron conductivity, leading to the waste of a large portion of catalysts and low catalyst utilization [18]. In addition, they also clarified that directly depositing catalysts on the LGDL to form gas diffusion electrodes (GDEs) is an efficient strategy to improve catalyst utilization with much lower catalyst loading. Moreover, the electrode design of GDE/PEM enables ionomer-free electrodes, thus avoiding the conductivity and stability issues from the mixture of ionomers [9, 19–21]. Notably, to develop high-efficiency PEMECs with low cost, more efforts are needed for the anode side due to sluggish reaction kinetics. So far, several anode GDEs have been reported for PEMECs. For example, via a sputtering method, IrO_2_ catalysts were deposited on carbon paper substrates by Slavcheva and co-authors [22] to serve as anodes for PEMECs. In addition, an Ir-based GDE was prepared for PEMECs by Jeong and co-authors [23] by combining electrochemical methods with subsequent oxidation processes, which resulted in 340 mA cm^−2^ at 1.6 V. Recently, instead of using traditional LGDLs, Yu and co-authors [24] adopted novel thin-tunable liquid/gas diffusion layers (TTLGDLs) as the LGDL to electrodeposit metallic iridium and demonstrated that the metallic iridium-coated electrode showed improved catalyst utilization compared to the commercial CCM design. Nevertheless, cell performances need further improvement relative to these studies. Electrodes with high intrinsically active catalysts, excellent stability and efficient and easily scalable fabrication are required in the commercialization of PEMECs.

In this study, amorphous IrO_*x*_ catalyst-coated TTLGDLs (IrO_*x*_ CCLGDLs) are first prepared as highly efficient anodes for PEMECs with low cost via a sustainable and easily scalable electrodeposition process at room temperature. With an ultralow loading of 0.075 mg cm^−2^, the fabricated amorphous IrO_*x*_ CCLGDL shows a high cell efficiency of about 90%, achieving more than 96% catalyst savings and 42-fold higher catalyst utilization compared to the commercial CCM design. Meanwhile, compared with most of the previously reported anodes for PEMECs, the IrO_*x*_ CCLGDL shows superior performance, improved catalyst utilization, and significantly simplified and scalable electrode fabrication, which promote the large-scale application of PEMECs. Notably, the remarkable performance is mainly due to the amorphous phase, rich OH groups, and sufficient Ir^3+^ content in catalysts, which could enable abundant active sites for the electrochemical reaction. Hence, the high-performance IrO_*x*_ CCLGDL could be applied to industry to accelerate PEMEC commercialization and renewable energy evolution. Meanwhile, the IrO_*x*_ CCLGDL could be easily extended to other electrochemical devices such as unitized regenerative fuel cells.

## Experimental Section

### Chemicals

Iridium (III) trichloride hydrate (IrCl_3_·*x*H_2_O, 99%), oxalic acid (OA, H_2_C_2_O_4_, crystalline 99.5%–102.5%), and potassium carbonate ((K_2_CO_3_, 99.5%) were purchased from Alfa Aesar and used as received. Deionized (DI) water with a resistivity of 18 ΜΩ cm was used.

### IrO_***x***_ CCLGDL Preparation

Thin/well-tunable LGDLs (TTLGDLs) with a thin thickness of 75 μm and porosity of 40% were used as the substrates to electrodeposit IrO_*x*_ catalysts. Before the electrodeposition process, the TTLGDLs were washed with methanol and acetone, respectively, and then treated in 0.05 M oxalic acid (OA) at 90 °C for 20 min to remove the surface Ti oxides. Afterward, a thin Au layer was sputtered on the TTLGDL to serve as a protection layer. To prepare the electrolyte, IrCl_3_·*x*H_2_O (4 mM) and oxalic acid (20 mM) were added into the DI water and stirred for about 30 min. Subsequently, potassium carbonate was added into the above solution to tune the pH value within 10–10.5. Afterward, the resultant solution was heated at 40 °C for 20 h to form hydrolyzed Ir^3+^/Ir^4+^ complexes. Subsequently, via a CV scan electrodeposition process within − 0.85 ~ 1 V vs*.* SCE with a scan rate of 50 mV s^−1^, IrO_*x*_ catalysts with different loadings of about 0.075, 0.170, and 0.340 mg cm^−2^ were electrodeposited on the TTLGDLs, which are noted as 0.075-based CCLGDL, 0.170-based CCLGDL, and 0.340-based CCLGDL, respectively.

### Characterization

A field emission JSM-IT700HR scanning electron microscope (SEM) with energy-dispersive X-ray spectroscopy (EDS) and Hitachi HF3300 transmission electron microscopy (TEM) was used to characterize the morphology and composition of the catalysts. A Rigaku SmartLab X-ray diffraction (XRD) system was used to analyze the catalyst's crystalline structure. With a Quantum 2000 Scanning ESCA Microprobe and a monochromatic Al K*α* X-ray source, the X-ray photoelectron spectroscopy (XPS) was conducted. Survey scans were collected between 0 and 1350 eV. All the peak shift corrections were done with C 1*s* at 284.8 eV. With an operation accelerating voltage of 80 kV, the material phase of IrO_*x*_ catalysts was studied by scanning transmission electron microscopy (STEM) on a probe-corrected JEOL NEOARM.

### PEMEC Assembly

A commercial CCM with 2.0 mg_Ir_ cm^−2^ IrO_x_ at the anode side and 1.0 mg_PtB_ cm^−2^ Pt black at the cathode side was used as a baseline. A single-side Nafion 117 CCM with 1.0 mg_PtB_ cm^−2^ at the cathode side (Nel Hydrogen, CT) and Toray 090 carbon papers were employed for the cell assembly. The prepared IrO_*x*_ CCLGDL was used as the anode. Along with gaskets, the N117 CCM was sandwiched between the carbon paper and the prepared IrO_*x*_ CCLGDL to form an MEA with an active area of 5 cm^2^. With graphite plate at the cathode side and Au-coated titanium plate at the anode side, the obtained configuration was sandwiched by two stainless steel endplates, tightening with eight evenly distributed bolts to achieve 40 in lb of torque to obtain a whole PEMEC.

### PEMEC Test

The assembled PEMEC was operated at 80 °C, with a water flow rate of 20 mL min^−1^ at the anode side, and under atmospheric pressure at both the anode and cathode sides. A Potentiostat VSP/VMP3B-100 (Bio-Logic) was adopted to record all data including the polarization curves, the electrochemical impedance spectroscopy (EIS) plots, and high-frequency resistance (HFR) plots. The EIS plots were recorded within a frequency range of 10 kHz–100 mHz under current densities of 0.2 A cm^−2^. The HFR plots were measured from 0 to 2 A cm^−2^ at 5 kHz. A commercial CCM with 2.0 mg cm^−2^ IrO_*x*_ at the anode and 1.0 mg cm^−2^ Pt black at the cathode was used as a baseline.

## Results and Discussion

### Characterization of the Amorphous IrO_*x*_ Thin Electrode

**Fig. 1:**
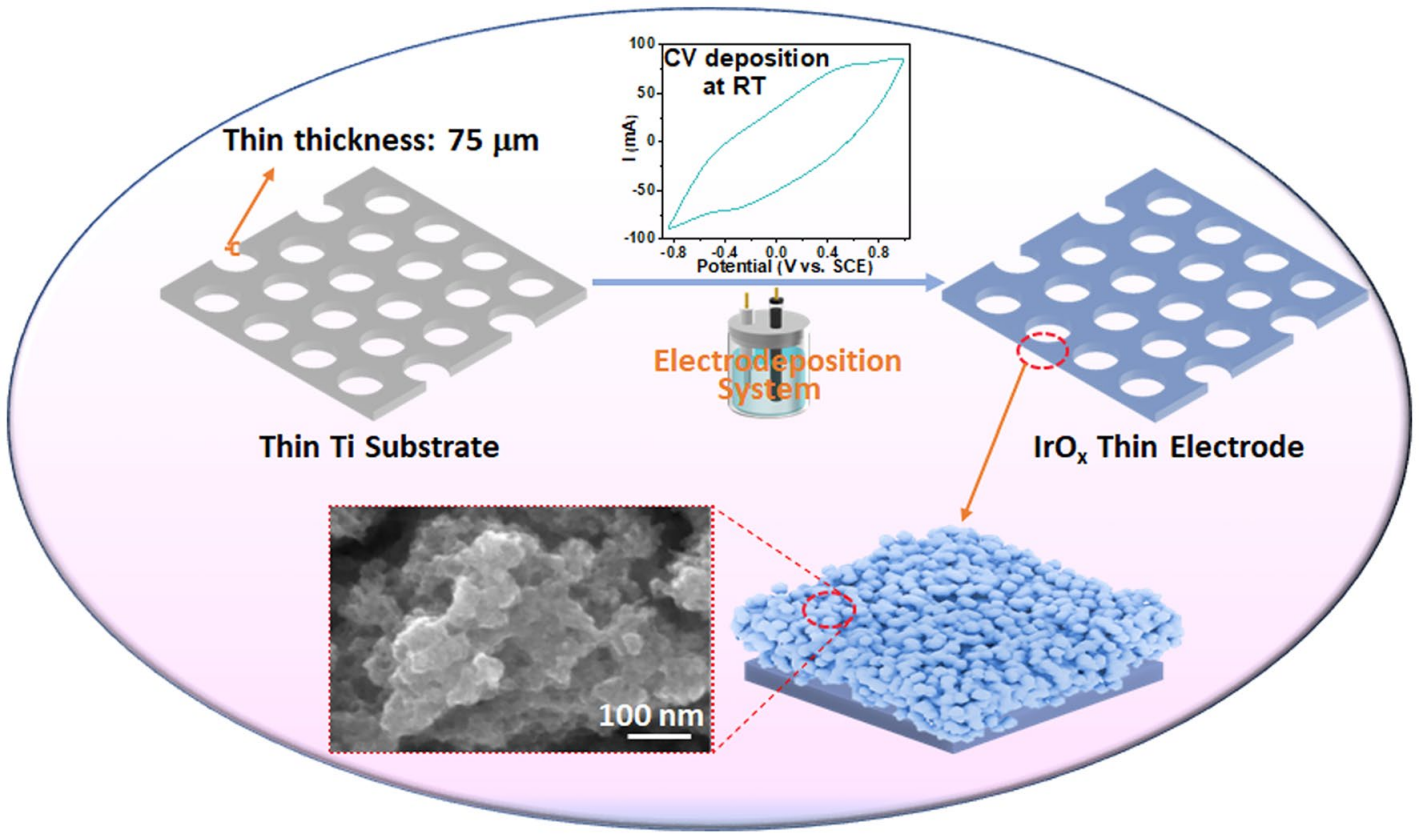
Schematic of the IrO_*x*_ catalyst-integrated thin electrode fabrication

As shown in Fig. 1, a thin Ti substrate of a TTLGDL is used to deposit IrO_*x*_ catalysts through an electrochemical method in a three-electrode system, in which a cyclic voltammetry (CV) electrodeposition process is conducted at room temperature and under ambient pressure. The electrodeposition process is facile with green and reusable electrolytes and no elaborate equipment involved, which is sustainable and easy to scale up to prepare large electrodes at low costs. Hence, it can be easily extended to industrial applications. Notably, compared to traditional LGDLs with thick thicknesses and 3D random structures, the nature of thin thickness and straightforward pore structures of TTLGDL permits good control of water/electron/thermal distribution to overcome the large interfacial contact resistances caused by random pore shapes and structures. In addition, the TTLGDL allows higher catalyst savings because of the planar surfaces. Therefore, the TTLGDL is adopted as novel substrate for preparing CCLGDLs with high catalyst utilization for high-efficiency electrolyzer cells [4, 10, 18, 25, 26]. For comparison, three catalyst loadings of 0.075, 0.170, and 0.340 mg cm^−2^ were deposited on the TTLGDLs by varying the deposition duration, forming different CCLGDLs.

**Fig. 2:**
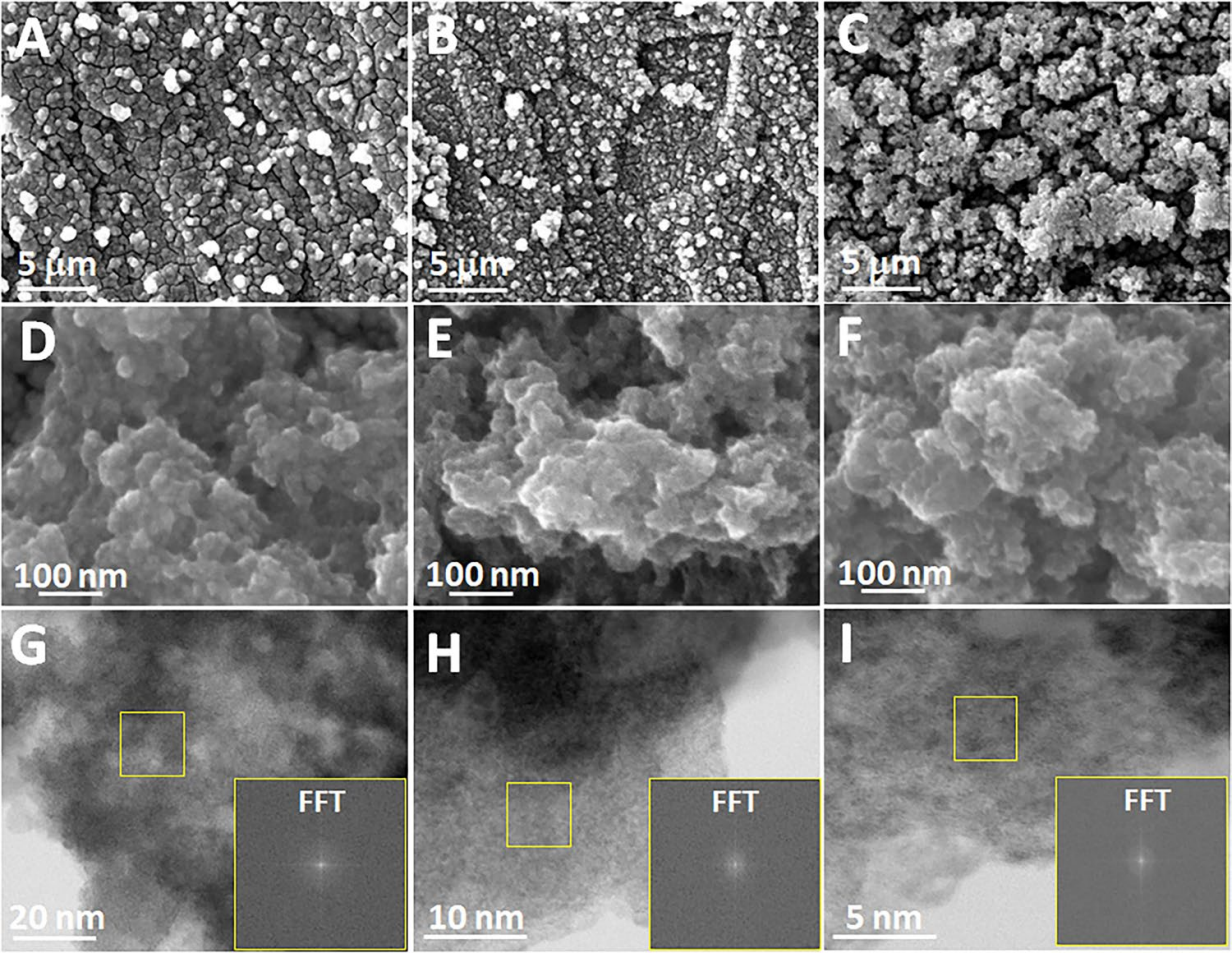
SEM images of IrO_*x*_ CCLGDLs with different loadings **A**, **D** 0.075 mg cm^**–**2^ IrO_*x*_; **B**, **E** 0.170 mg cm^**–**2^ IrO_*x*_; **C**, **F** 0.340 mg cm^**–**2^ IrO_*x*_. **G–I** High-angle annular dark-field (HAADF) STEM images at different magnifications and the related FFT pattern of the IrO_*x*_ catalysts

The obtained CCLGDLs were characterized by the SEM to investigate catalyst layer morphologies. As shown in Fig. S1, before the IrO_*x*_ catalyst deposition, the bare TTLGDL exhibits smooth surfaces. With the catalyst deposition, various catalyst layer morphologies are presented for the different CCLGDLs with different IrO_*x*_ loadings, showing increasing roughness as the catalyst loading rises (Fig. 2A–C). At a higher magnification, the IrO_x_ catalysts exhibit small nanoparticle morphologies with porous structures and rough surfaces for all loading-based samples, which are expected to expose abundant active sites for oxygen evolution reactions (OERs) (Fig. 2D–F). In addition, SEM–EDS mapping and EDS characterizations were performed to investigate the element distribution, surface coverage, and element content on the TTLGDLs. As shown in Figs. S2–S4, the Ir element is uniformly distributed on the TTLGDL substrate surface, exhibiting atomic ratios of 3.09%, 6.71%, and 13.48%, for the 0.075-based CCLGDL, 0.170-based CCLGDL, and 0.340-based CCLGDL, respectively. Meanwhile, an oxygen element with good coverage on the substrate is also detected, which could reveal that the catalysts show an oxidation state rather than metallic iridium. Notably, the oxygen contents might be also from the absorbed water in the catalysts and the SEM sample stage.

To gain more insights into the deposited IrO_*x*_ catalysts, high-angle annular dark-field scanning transmission electron microscopy (HAADF-STEM) measurements at different areas were also performed. As shown in Fig. 2G–I, the HAADF-STEM images with Fourier transform patterns inserted demonstrate that the deposited IrO_*x*_ catalysts show an amorphous phase, which is expected to expose rich active sites for the electrochemical reaction. Previous studies also confirmed the amorphous phase of the prepared materials via high-resolution transmission electron microscopy (HRTEM) along with Fourier transforms [27–29]. Amorphous materials have rich randomly oriented bonds and unsaturated sites with high density, which could enhance the reactant adsorption and thus improve catalyst activity [30–33]. Some previously reported studies also demonstrated the advantage of amorphous materials. For example, Mu et al. also adopted amorphous MoO_*x*_^3−^ as matrixes to stabilize Ru single-atoms to achieve excellent performances for both HER and OER. Part of the reasons for the excellent performances are due to the amorphous matrixes, which can provide abundant defects for the reaction and then deliver better performance compared to the crystalline materials [32]. Additionally, Mu et al. reported that the amorphous VO_*x*_ in the crystalline/amorphous-Ru/VO_*x*_ heterogeneous catalysts can expose more active sites, enhance charge transport, and then achieve improved electronic interaction between the metal and the support [33]. Moreover, XRD characterizations were conducted on both the IrO_*x*_/Ti sample and the IrO_*x*_/carbon paper (CP) sample to further investigate the IrO_*x*_ catalyst crystal structure. As shown in Figs. S5and S6, apart from peaks assigned to the Ti and CP substrate, there are no peaks assigned to IrO_*x*_ detected, which further validates the amorphous phase of the IrO_*x*_ catalysts. Additionally, the whole IrO_*x*_ CCLGDL cross section is characterized via the SEM to check the electrode thickness. As shown in Fig. S7, the CCLGDL thickness is about 75 µm, much thinner than the conventional LGDLs such as the conventional Ti felt and sintered Ti powder (up to 350 µm). Notably, the thin CCLGDL is weight, material, and volume-saving, which could decrease the PEMEC cost and accelerate its large-scale application.

**Fig. 3:**
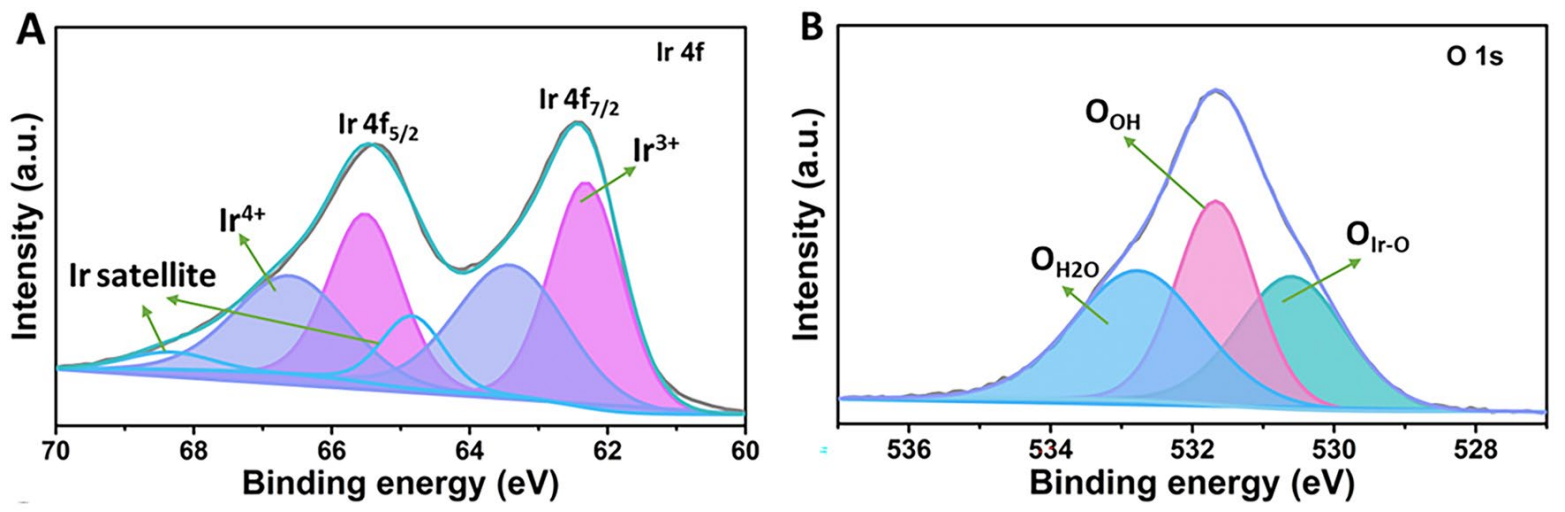
**A** Ir XPS spectra and **B** O XPS spectra of the fresh IrO_*x*_ catalysts

To investigate the surface composition, chemical state, and electrical structure of the catalysts, XPS characterization was employed. As shown in Fig. 3A, after the peak splitting, peaks assigned to Ir^3+^ and Ir^4+^ with two Ir satellite peaks are observed in the Ir species. Specifically, there are two peaks of Ir^4+^ at 63.40 eV for Ir^4+^ 4*f*_7/2_ and 66.60 eV for Ir^4+^ 4*f*_5/2_, respectively, showing an Ir^4+^ content of 47.41%. While two peaks at 62.31 eV and 65.51 eV are ascribed to Ir^3+^ 4*f*_7/2_ and Ir^3+^ 4*f*_5/2_, respectively, exhibiting a higher Ir^3+^ content of 52.59% compared to that of Ir^4+^. The higher Ir^3+^ content in the amorphous IrO_*x*_ catalyst could enhance the catalytic activity due to rich electrophilic oxygen O^−^, which could cause water nucleophilic attack, as reported by previous studies [34–36]. Some in situ characterization techniques have been used to study the reaction behavior and pathway of the Ir-based materials so far. For example, Pfeifer et al*.* [34] demonstrated the existence and formation of electrophilic oxygen O^−^ species in an amorphous and mixed-valent iridium matrix during the OER process using in situ soft X-ray absorption spectroscopy (sXAS) and X-ray photoemission (XPS). They also found there was a positive correlation between the generated O_2_ and the concentration of the electrophilic oxygen O^−^, for instance, the O^−^ concentration increased during the oxygen evolution activity measurement and disappeared without the potential application. Frei et al*.* [35] detected Ir-OOH species with O–O vibration at 830 cm^−1^ in IrO_*x*_ clusters by using rapid scan Fourier transform infrared spectroscopy (FTIR) under visible light and investigated the material behavior via in situ Raman spectroscopy under the OER condition. In future research, some in situ characterization techniques such as in situ XAS, in situ Raman and in situ FTIR will also be used to understand the excellent catalytic performance and reaction mechanism in our studies if they are accessible. Notably, satellite peaks are also observed in the Ir XPS spectra, which are from a sudden change in Coulombic potential as the photo-ejected electron passes through the valence band. When ejected electrons lose some energy due to other electronic and magnetic processes, and as a result, energy different from their original binding energy occurs and generates satellite peaks [37, 38]. Different species and oxidation states have different contributions to satellite peaks. The satellite peaks might affect the performance of the material [37, 38]. For the O 1*s* XPS spectra (Fig. 3B), three peaks are presented, a peak at 530.60 eV corresponding to the lattice oxygen in the Ir-O-Ir bond, a peak at 531.66 eV ascribed to the hydroxyl oxygen, and a peak at 532.94 eV from the oxygen in the adsorbed water, showing contents of 30.3%, 34.3%, and 35.5%, respectively. Notably, compared to the lattice oxygen, hydroxyl oxygen exhibits a higher content, demonstrating more OH groups on the catalyst surface, which is beneficial for improving the OER performance [39, 40]. The OH groups can be converted into electrophilic O^−^ species (IrO_*x*_O^2−^H → IrO_*x*_O^−^  + H^+^  + e^−^), which tend to access H_2_O or hydroxyl species and then promote the O–O bond formation. In addition, the surface OH groups enable moderate oxygen intermediate binding energies since lattice oxygen shows stronger intermediate binding than the OH group [41, 42].

### Performance Evaluation in PEMECs

**Fig. 4:**
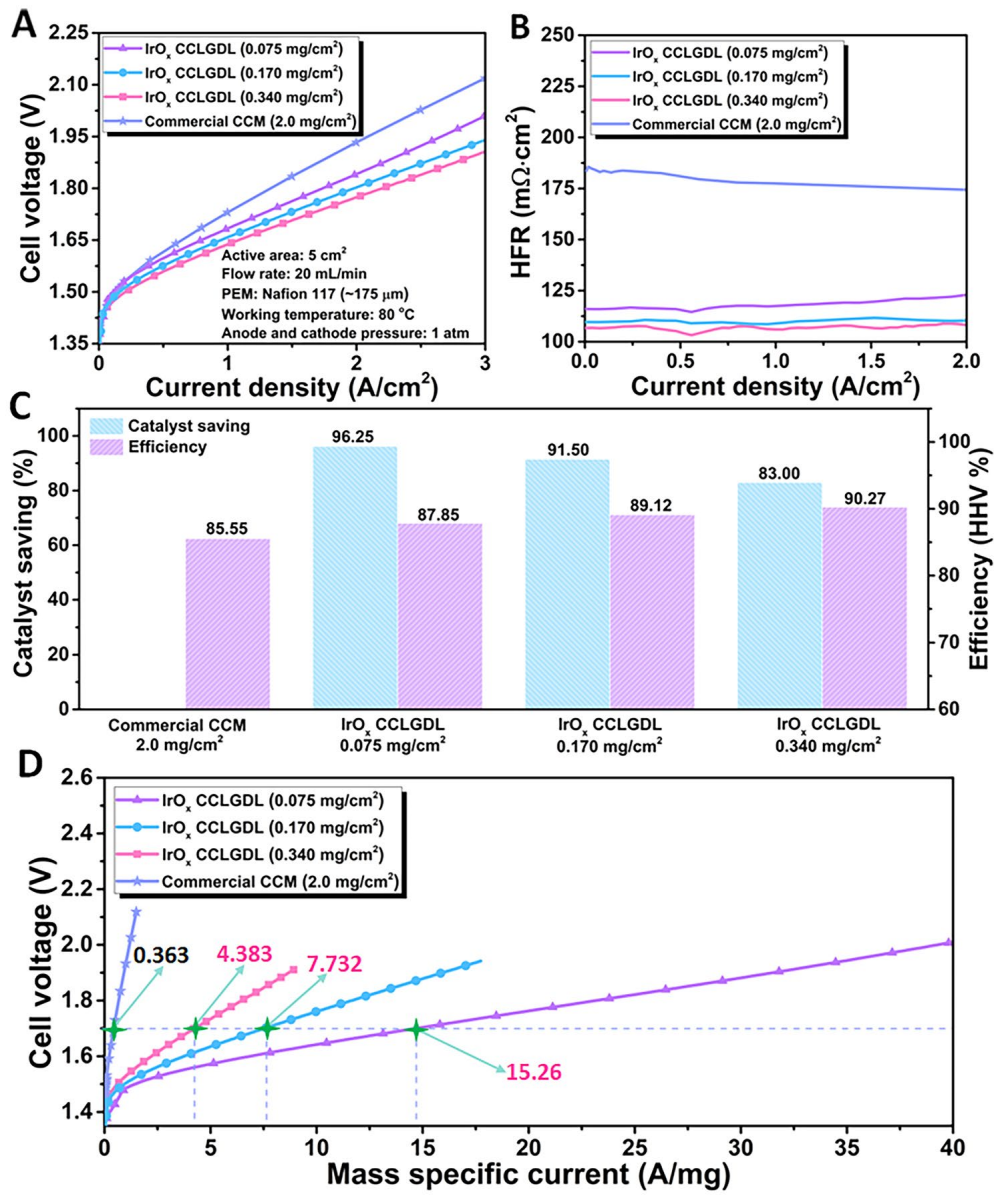
**A** Cell polarization curves; **B** the related high frequency resistance (HFR) plots; **C** Catalyst saving and efficiency comparison at 1 A cm^**–**2^. **D** Ir mass normalized cell polarization curves

By combining with N117 membranes, cell performances of different IrO_*x*_ CCLGDLs were evaluated in the PEMECs at 80 °C and compared with the commercial CCM. As shown in Fig. 4A, compared to the CCM baseline, superior cell performances are achieved for all amorphous IrO_*x*_ CCLGDLs, showing a high current operation of up to 3 A cm^−2^ with low cell voltages ≤ 2.00 V. Specifically, a low cell voltage of only 1.77 V at 2 A cm^−2^ is demonstrated for the 0.340-based CCLGDL, which is 160 mV lower than that of the commercial CCM (1.93 V). In addition, the 0.340-based CCLGDL also delivers a low cell voltage of 1.91 V at a current density of 3 A cm^−2^, while for the CCM baseline, a high cell voltage of 2.12 V is observed at the current density of 3 A cm^−2^. For the other two catalyst loadings of 0.170 and 0.075 mg cm^−2^, superior performances are also observed compared to the CCM baseline, delivering low cell voltages of 1.84 and 1.80 V at 2 A cm^−2^, respectively. In addition, cell voltages ≤ 2.00 V are achieved at the current density of 3 A cm^−2^, showing 1.94 V for the 0.170-based CCLGDL and 2.00 V for the 0.075-based CCLGDL, respectively. Moreover, due to the ionomer-free IrO_*x*_ catalyst layer, the amorphous IrO_*x*_ CCLGDL shows much lower average HFR values of 107, 110, and 119 mΩ cm^2^ for the 0.340-based CCLGDL, 0.170-based CCLGDL and 0.075-based CCLGDL, respectively (Fig. 4B). In contrast, the commercial CCM exhibits a high average HFR value of 180 mΩ cm^2^, which is mainly ascribed to the ionomer-mixed catalyst layer with limited electron conductivity. Notably, similar average HFR values are observed for the 0.340-based CCLGDL and 00.170-based CCLGDL, while a little bit higher average HFR value is observed for the 0.075-based CCLGDL. This should be because the catalyst loading of 0.075-based CCLGDL is much lower than the other two samples and its catalyst layer surface is smoother. The smoother catalyst layer surface could affect the active reaction site number for the electrochemical reaction and thus affect the HFR value. Similar phenomena were also observed in previous studies, indicating that more reaction sites enable lower ohmic resistances. For example, Kang and co-authors considered reaction sites parallel with each other in the circuit and demonstrated that a lower ohmic loss was obtained with more reaction sites offered [43]. Additionally, by applying different TTLGDL patterns with various pore sizes, land widths and porosities, Shu and co-authors found that larger reaction site numbers would reduce the ohmic loss in the PEMEC and achieve a lower HFR value [24].

As illustrated in Fig. 4C, compared to the commercial CCM with a high anode catalyst loading of 2.0 mg cm^−2^, the CCLGDLs developed in our study save a lot of anode catalysts for the PEMEC. Specifically, about 96.25%, 91.50%, and 83.00% anode catalysts are saved for the 0.075-based CCLGDL, 0.170-based CCLGDL and 0.340-based CCLGDL, showing higher voltage efficiencies of 87.85%, 88.67%, and 90.27% at 1 A cm^−2^, respectively. While the commercial CCM presents a lower voltage efficiency of 85.55% at the current density of 1 A cm^−2^. Overall, the superior performances and efficiencies with much lower catalyst loadings demonstrate high catalyst utilization for the amorphous IrO_*x*_ CCLGDLs. To further validate the high catalyst utilization of the amorphous IrO_*x*_-integrated CCLGDL, cell voltages with current density normalized to Ir mass are shown in Fig. 4D. It is clearly seen that within the same testing current density range of 0–3 A cm^−2^, the 0.075-based CCLGDL, 0.170-based CCLGDL and 0.340-based CCLGDL can reach up to high mass specific currents of 40.06, 20.10, and 8.910 A mg^−1^, respectively, as shown in Table 1. In contrast, a low mass specific current of about 1 A/mg is observed for the commercial CCM. Moreover, to better demonstrate the high catalyst utilization of the amorphous IrO_*x*_ CCLGDLs and compare with recently reported anode catalysts, mass specific currents at a voltage of 1.7 V are collected and listed in Table 1. High mass specific currents of 15.26, 7.732, and 4.383 A mg^−1^ are delivered for the 0.075-based CCLGDL, 0.170-based CCLGDL, and 0.340-based CCLGDL, respectively, which are about 42.04, 21.30, and 12.07-fold higher than that of the commercial CCM (0.3630 A mg^−1^). The high catalyst utilization of the amorphous IrO_*x*_ CCLGLD is due to the ionomer-free catalyst layer with good conductivity and also the rough catalyst surfaces and high intrinsic activity of the amorphous IrO_*x*_ catalysts.

**Table 1:** Mass specific current comparison of IrO_x_ CCLGDLs with the commercial CCM

Electrode	Mass specific current at 1.7 V (A mg^−1^)	Increased times	Mass specific current within 0–3 A cm^−2^ (A mg^−1^)
0.075-based	15.26	42.04	0–40.06
0.170-based	8.892	21.30	0–20.10
0.340-based	4.383	12.07	0–8.910
CCM	0.363	–	0–0.9996

Meanwhile, for comparison, the cell performance of the TTLGDL combined with the commercial full CCM was also performed. As shown in Fig. S8A, the commercial CCM/TTLGDL design requires a higher cell voltage of 1.88 V at 2 A cm^−2^, which is inferior to all IrO_*x*_ CCLGDLs (< 1.84 V). Meanwhile, the average HFR value of the commercial CCM/TTLGDL design is about 143 mΩ cm^2^, which is also higher than those of all IrO_*x*_ CCLGDLs (< 119 mΩ cm^2^). Additionally, the Ir mass normalized polarization curve shown in Fig. S8B presents that the mass specific current at 1.7 V for the commercial CCM/TTLGDL is only 0.5400 A mg^−1^. This value is also much lower than those of all IrO_*x*_ CCLGDLs (≥ 4.383 A mg^−1^). These results further demonstrate the advantages of the ionomer-free IrO_*x*_ CCLGDL design, namely significantly decreased catalyst loading, high catalytic activity and cell performance, and remarkably improved catalyst utilization.

**Fig. 5:**
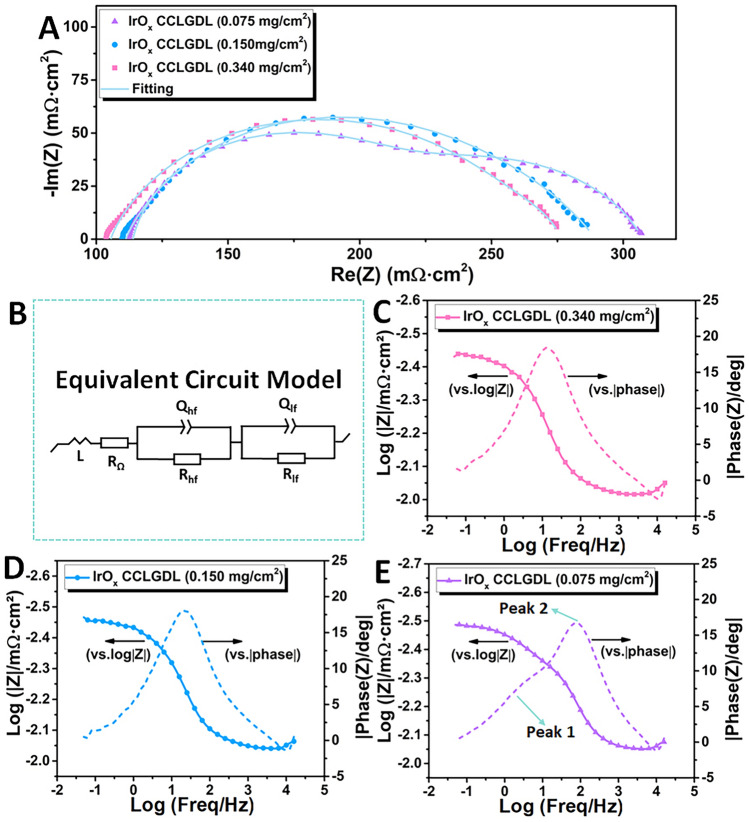
**A** EIS plots recorded at 0.2 A cm^**–**2^; **B** the related equivalent circuit model; **C** Bode plots of the 0.340-based CCLGDL; **D** Bode plots of the 0.170-based CCLGDL; **E** Bode plots of the 0.075-based CCLGDL

As discussed above, the fabricated amorphous IrO_*x*_ CCLGDLs show superior performance than the commercial CCM while different performances are observed for CCLGDLs with different IrO_*x*_ loadings. Based on the SEM images shown in Fig. 2, we could induce that the different performances are mainly ascribed to the different catalyst layer morphologies. The rougher catalyst layer surface is observed with the catalyst loading increase, which can expose more active sites for the electrochemical reaction. This should be the main reason for the better performance with higher catalyst loading. To validate the assumption, EIS plots of different CCLGDLs recorded at 0.2 A cm^−2^ were collected (Fig. 5A) and statistically analyzed based on an appropriate equivalence circuit model (Fig. 5B), which can provide insights into ohmic loss, activation loss and double-layer capacitances related to reaction site number. As shown in Fig. 5A, a distorted semi-arc is observed for both 0.340-based CCLGDL and 0.170-based CCLGDL, which is ascribed to two overlapping semi-arcs at the high frequency (HF) range and low frequency (LF) range, respectively, indicating a two-time constant process with similar time constants. Thus, two overlapping peaks are observed in the related Bode plots, as shown in Fig. 5C, D. For the 0.075-based CCLGDL, two semi-arcs are presented and the semi-arc at the LF range is obvious, implying a two-time constant process with different time constants corresponding to the phase shift in the Bode plots (Fig. 5E). Meanwhile, two obvious peaks are presented in the Bode plots. Hence, the semi-arc at the LF range becomes more obvious as the catalyst loading decreases from 0.340 to 0.075 mg cm^−2^, which should be due to decreased reaction sites, and a similar phenomenon is also in previous studies [10].

To statistically analyze the EIS results, the EIS plots were fitted based on the equivalence circuit model, which involves an inductor (*L*), an ohmic resistance (*R*_Ω_) connected in series, and two pairs of resistance in parallel with a constant phase element assigned to the HF (*R*_hf_ and *Q*_hf_) and LF (*R*_lf_ and *Q*_lf_) range, respectively. As listed in Table 2, ohmic resistances of about 103, 109, and 119 mΩ cm^2^ are observed for the 0.340-based CCLGDL, 0.170-based CCLGDL and 0.075-based CCLGDL, respectively, showing a similar trend to the HFR results. Since the EIS plots were recorded at a low current density of 0.2 A cm^−2^, the two semi-arcs should be from the charge transfer process and mass transport should not be an issue. The total resistances from the two semi-arcs represent the total activation loss of the PEMEC. As presented in Table 2, the total resistance ascribed to the activation loss increases as the catalyst loading decreases, showing about 171, 180, and 191 mΩ cm^2^ as the catalyst loading decreases from 0.340 to 0.075 mg cm^−2^. Meanwhile, the double-layer capacitance (*C*_dl_) between the CCLGDL interface and the PEM is also derived, which can represent active sites for the reaction. As shown in Table 2, double-layer capacitance values at the HF (*C*_dl, hf_) and LF (*C*_dl, lf_) ranges are both derived and compared for the three CCLGDLs. With more active sites exposed, a higher *C*_dl, hf_ value at the HF range could be achieved [30, 31, 44]. Notably, the *C*_dl, lf_ at the LF range is considered as a “slow process” since a larger time constant is needed for the reaction sites [26]. Hence, different from the *C*_dl, hf_, a smaller *C*_dl, lf_ is desired for the catalyst/electrode design. With a smaller *C*_dl, lf_ value, less reaction sites would be involved in a larger time constant. Overall, the *C*_dl, hf_ value increased along with the IrO_*x*_ loading increased. Specifically, the 0.340-based CCLGDL shows the highest *C*_dl, hf_ value of 164.3 mF cm^−2^, and the 0.075-based CCLGDL delivered the lowest *C*_dl, hf_ value of 29.56 mF cm^−2^. The 0.17-based CCLGDL shows a moderate* C*_dl, hf_ value of 146.0 mF cm^−2^. While the *C*_dl, lf_ values presented an opposite trend: the *C*_dl, lf_ value decreased along with the IrO_*x*_ loading increased. These results are reasonable, since as shown in the SEM images in Fig. 2, the surface roughness increased along with the catalyst loading increased, which can offer a higher surface area and expose more active sites for the reaction. As a result, inferior performances are presented for the 0.075-based CCLGDL and 0.17-based CCLGDL compared to the 0.34-based CCLGDL.

**Table 2:** The EIS fitting data derived from Fig. 5A

CCLGDL	*R*_Ω_ (mΩ cm^2^)	*R*_hf_ (mΩ cm^2^)	*R*_lf_ (mΩ cm^2^)	*R*_total_ (mΩ cm^2^)	*C*_dl, hf_ (mF cm^−2^)	*C*_dl, lf_ (mF cm^−2^)	Error
0.075-based	119.4	79.30	111.8	191.1	29.56	324.8	0.2568
0.170-based	109.0	40.02	140.1	180.1	146.0	247.6	0.3916
0.340-based	102.6	1.211	169.8	171.0	164.3	141.4	0.4223

**Fig. 6:**
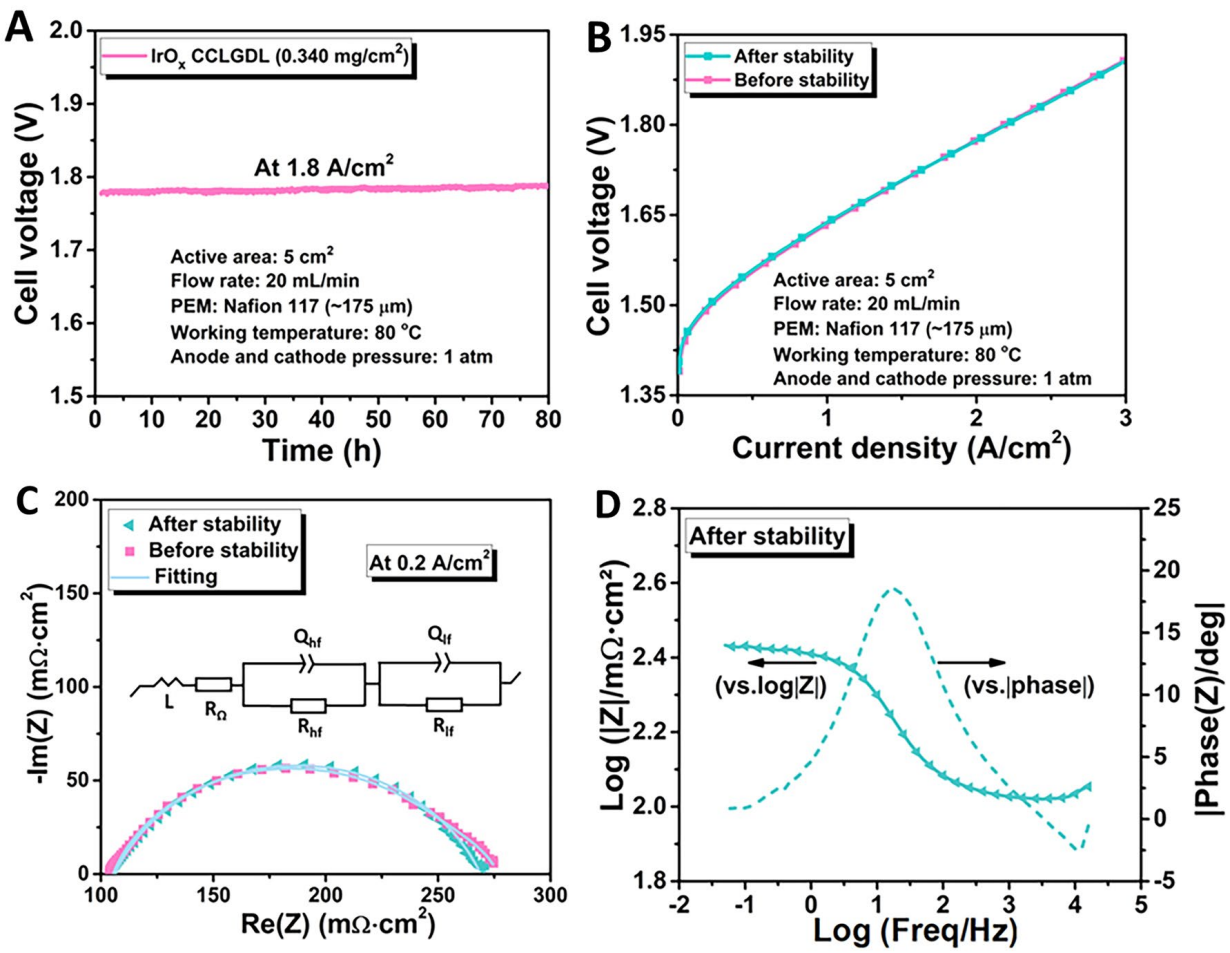
**A** Cell stability test at 1.8 A cm^**–**2^. **B** Cell polarization curves of the IrO_x_ CCLGDL before and after the stability test. **C** EIS plots of the IrO_*x*_ CCLGDL before and after the stability test and **D** Bode plots of the tested IrO_*x*_ CCLGDL

Moreover, the excellent stability of the IrO_*x*_ CCLGDL was demonstrated at a current density of 1.8 A cm^−2^. As shown in Fig. 6A, the assembled PEMEC based on 0.340-based CCLGDL delivers a low degradation rate of 0.104 mV h^−1^ after operating at 1.8 A cm^−2^ for 80 h. Notably, the degradation is recoverable and polarization curves before and after the stability test were found to overlap with each other (Fig. 6B). The recoverable performance could be related to gas accumulation within the catalyst layer or the interphase between the catalyst layer and the membrane. A similar phenomenon was also observed in previous studies [5, 10, 25]. The accumulated gas can lead to some voltage efficiency loss. However, the performance loss can be mitigated by some efficient strategies in future research. For example, developing some nanostructured catalyst layers would mitigate the gas accumulation and performance loss due to the promoted bubble release and transport, which would mitigate the gas accumulation and performance loss as a result [4, 10, 12, 21]. In addition, creating some microchannels or pore-gradient structures on the gas diffusion layer is also a potential strategy to enhance the water flow and bubble release [6, 25]. To further investigate electrochemical property changes of the tested IrO_x_ CCLGDL, the EIS curves recorded at 0.2 A cm^−2^ before and after the stability test are compared. As shown in the EIS plots (Fig. 6C), the two EIS plots almost overlapped, and based on the equivalence circuit model, the plots were fitted. As shown in Table S1, the *R*_Ω_ value is maintained (102.6 mΩ cm^2^) before and after the stability test, matching well with the HFR results (Fig. S9). Meanwhile, similar charge transfer resistance values are observed, showing total values of about 171 and 169 mΩ cm^2^, respectively. In addition, similar double-layer capacitances at HF (*C*_dl, hf_) are also obtained (before the stability test: about 164 mF cm^−2^ and after the stability test: about 166 mF cm^−2^), indicating that active electrochemical reaction sites are maintained after the stability test. Meanwhile, two overlapping peaks are also presented in the related Bode plots after the stability test (Fig. 6D). Overall, after the stability test, the electrochemical properties of active reaction sites, ohmic resistance, and charge transfer resistance of the IrO_*x*_ CCLGDL are maintained, resulting in similar cell performance after the 80-h stability test at the current density of 1.8 A cm^−2^.

### Post-Analysis After the Stability Test in PEMECs

**Fig. 7:**
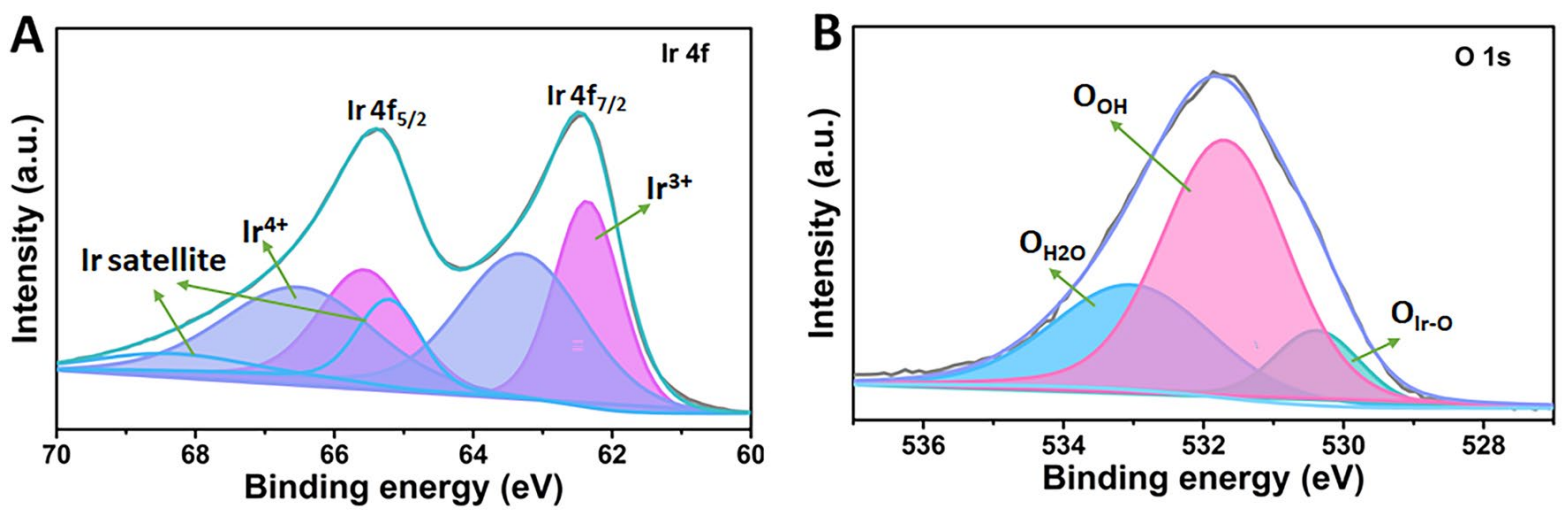
**A** Ir XPS spectra and **B** O XPS spectra of the tested IrO_x_

Meanwhile, XPS analysis of the tested IrO_*x*_ was also conducted and compared to that of the fresh IrO_*x*_ to investigate the surface composition change (Table 3). Similar to the Ir XPS spectra of the fresh IrO_x_, peaks assigned to Ir^3+^, Ir^4+^, and satellite are observed after the peak splitting (Fig. 7A). As shown in Table 3, compared to the fresh IrO_x_ catalysts, decreased Ir^3+^ content (47.15%) is observed, while the Ir^4+^ content increases (52.85%), which should be due to the transformation of Ir^3+^ to Ir^4+^ during the stability test process. The decreased Ir^3+^ content is reasonable owing to the high-anodic operation conditions at the anode side. In Fig. 7B, the XPS result for O 2*p* shows a higher content of hydroxyl (63.3%) in the tested IrO_*x*_ compared to that of the fresh IrO_*x*_ (34.3%), indicating there is more availability and capability for the formation of the O–O bond for OERs.

**Table 3:** XPS data comparison of the IrO_*x*_ before and after the 80 h stability test at 1.8 A cm^**–**2^

Samples	Ir			O		
	composition	Peak position (eV)	Atomic ratio (%)	Composition	Peak position (eV)	Atomic ratio (%)
Fresh IrO_*x*_	Ir^3+^	4*f*_7/2_: 62.31; 4*f*_5/2_: 65.51	52.59	O_Ir-O_	530.60	30.25
	Ir^4+^	4*f*_7/2_: 63.40; 4*f*_5/2_: 66.60	47.41	O_OH_	531.66	34.29
				O_H2O_	532.94	35.46
Tested IrO_*x*_	Ir^3+^	4*f*_7/2_: 62.34; 4*f*_5/2_: 65.54	47.15	O_Ir-O_	530.85	15.62
	Ir^4+^	4*f*_7/2_: 63.37; 4*f*_5/2_: 66.57	52.85	O_OH_	531.93	63.31
				O_H2O_	532.92	21.07

### Performance and Catalyst Utilization Comparison

**Fig. 8:**
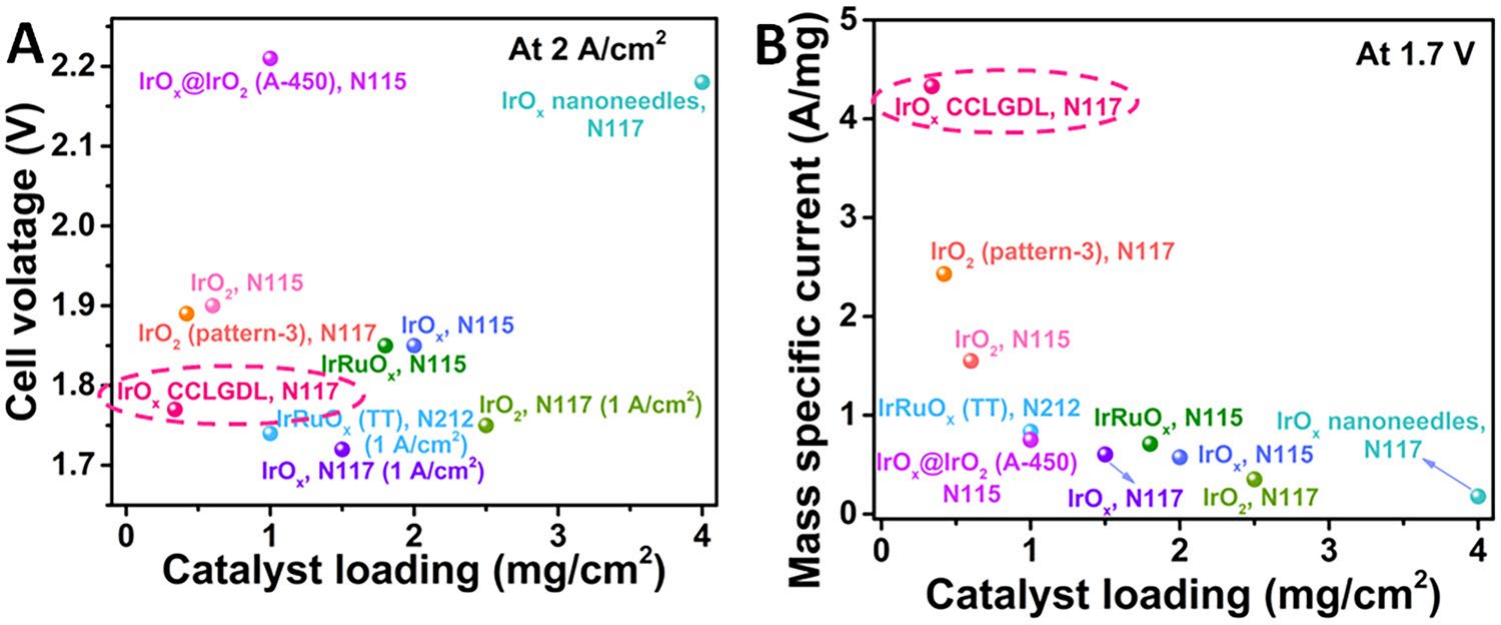
**A** Cell voltage comparison at 2 A cm^**–**2^ with previously reported anode catalysts: IrO_2_ (pattern-3), N117 [45]; IrO_2_, N117 [46]; IrRuO_*x*_, N115 [47]; IrO_2_, N115 [47]; IrO_2_ nanoneedles, N117 [48]; IrO_2_, N117 [49]; Ir_0.7_Ru_0.3_O_*x*_(TT), N212 [50]; IrO_*x*_@IrO_2_(A-450), N115 [51]; IrO_2_, N115 [52]; **B** the corresponding mass activity comparison at 1.7 V

To illustrate the remarkable performance and significantly simplified fabrication of the IrO_x_ CCLGDL, detailed cell test properties with fabrication methods of different previously reported anodes in PEMECs are collected and compared in Table S2. As shown in Fig. 8, compared with various anodes, the IrO_*x*_ CCLGDLs achieve low cell voltages of 1.77, 1.80, and 1.84 V at 2 A cm^−2^ with low catalyst loadings of 0.340, 0.170, and 0.075 mg cm^−2^ even with thick N117 membranes. In addition, high mass specific currents of 15.26, 7.732, and 4.383 A mg^−1^ at 1.7 V are delivered for the 0.075-based CCLGDL, 0.170-based CCLGDL, and 0.340-based CCLGDL, respectively, which are much higher than other anode catalysts, indicating a high catalyst utilization of the CCLGDLs. Moreover, for the electrode fabrication, instead of using conventional methods of spray or decal, which normally involves a complex fabrication process with multiple steps and elaborate equipment and thick electrodes with thick catalyst layers, a significantly simplified fabrication process of facile one-step electrodeposition process at room temperature is used to fabricate the ionomer-free thin IrO_*x*_ CCLGDL, which shows low cost, easy scalability and high efficiency. Overall, the facile and significantly simplified fabrication process enables an easy scale-up of large electrode fabrication with high efficiency, low cost, and sustainability, which could accelerate the large-scale application of the PEMEC.

## Conclusions

In this study, highly active amorphous IrO_*x*_-integrated thin electrodes are first developed as high-efficiency anodes for PEMECs through a facile, low-cost and scalable electrodeposition process at room temperature. Due to the amorphous phase, the reasonable Ir^3+^ content and rich surface OH groups, the IrO_*x*_ thin electrode delivers a high cell efficiency of about 90% with an ultralow loading of 0.075 mg cm^−2^ when assembled with an N117 membrane, achieving more than 96% catalyst savings and 42-fold catalyst utilization compared to the CCM baseline. Additionally, compared with the most previously reported anodes, the IrO_*x*_ electrode not only achieves better performance and higher catalyst utilization but also presents an easily scalable and significantly simplified fabrication process. Hence, the IrO_*x*_ thin electrode exhibits great potential to be applied to industry to accelerate PEMEC commercialization and could be easily extended to other electrochemical energy conversion and storage devices.

## Supplementary Information

Below is the link to the electronic supplementary material.

Supplementary file1 (DOCX 2843 KB)
