# Supplementary material for: Amorphous Iridium Oxide-Integrated Anode Electrodes with Ultrahigh Material Utilization for Hydrogen Production at Industrial Current Densities
Source: Nanomicro Lett. 2024 May 24;16:203. doi: 10.1007/s40820-024-01411-7 (PMC11126398; doi:10.1007/s40820-024-01411-7)
Supplement: Supplementary file 1 — Supplementary file1 (DOCX 2843 KB) [file 40820_2024_1411_MOESM1_ESM.docx]

Supporting Information for

**Amorphous Iridium Oxide Integrated Anode Electrodes with Ultrahigh Material Utilization for Hydrogen Production at Industrial Current Densities**

Lei Ding^1, #^, Kui Li^1, #^, Weitian Wang^1^, Zhiqiang Xie^1^, Shule Yu^1^, Haoran Yu^2^, David A. Cullen^2^, Alex Keane^3^, Kathy Ayers^3^, Christopher B. Capuano^3^, Fangyuan Liu^4^, Pu-Xian Gao^4,5^, and Feng-Yuan Zhang^1,^*

^1^ Department of Mechanical, Aerospace & Biomedical Engineering, University of Tennessee, Knoxville, TN, 37996, USA

^2^ Center for Nanophase Materials Sciences, Oak Ridge National Lab, Oak Ridge, TN 37831, USA

^3^ Nel Hydrogen, Wallingford, CT 06492, USA

^4^ Institute of Materials Science, University of Connecticut, Storrs, CT 06269, USA

^5^ Department of Materials Science & Engineering, University of Connecticut, Storrs, CT 06269, USA

^#^ Lei Ding and Kui Li contribute equally to this work.

*Corresponding author. E-mail: [fzhang@utk.edu](mailto:fzhang@utk.edu) (Feng-Yuan Zhang)

**Supplementary Figures and Tables**


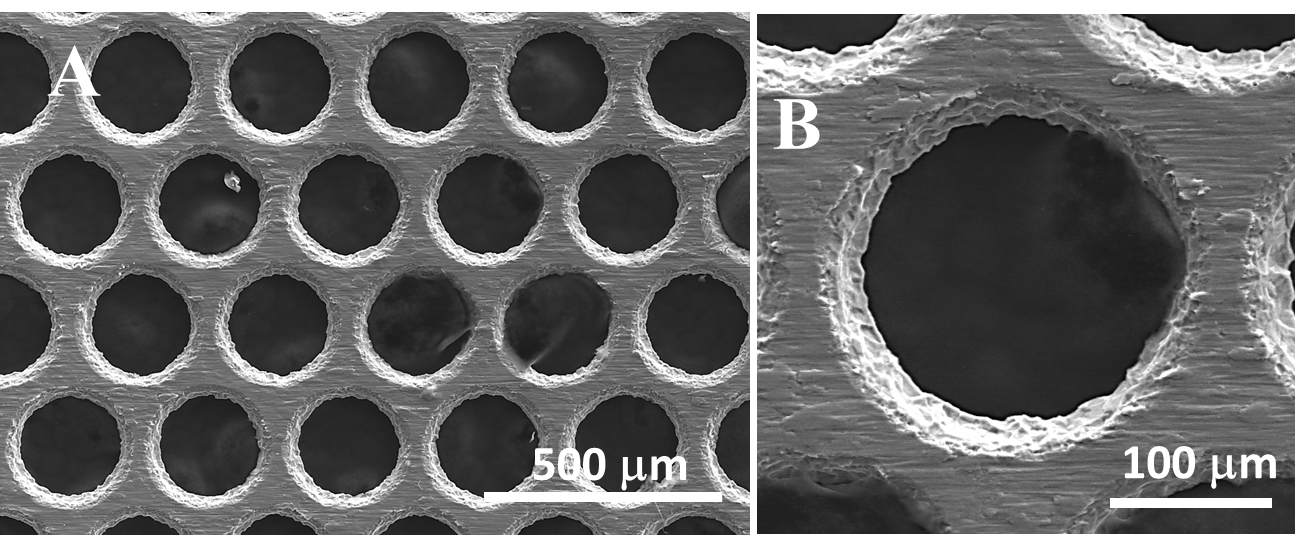


**Fig. S1** Top-view SEM image (A, B) of the TTLGDL with a pore size of 200 μm and a porosity of 40%

**
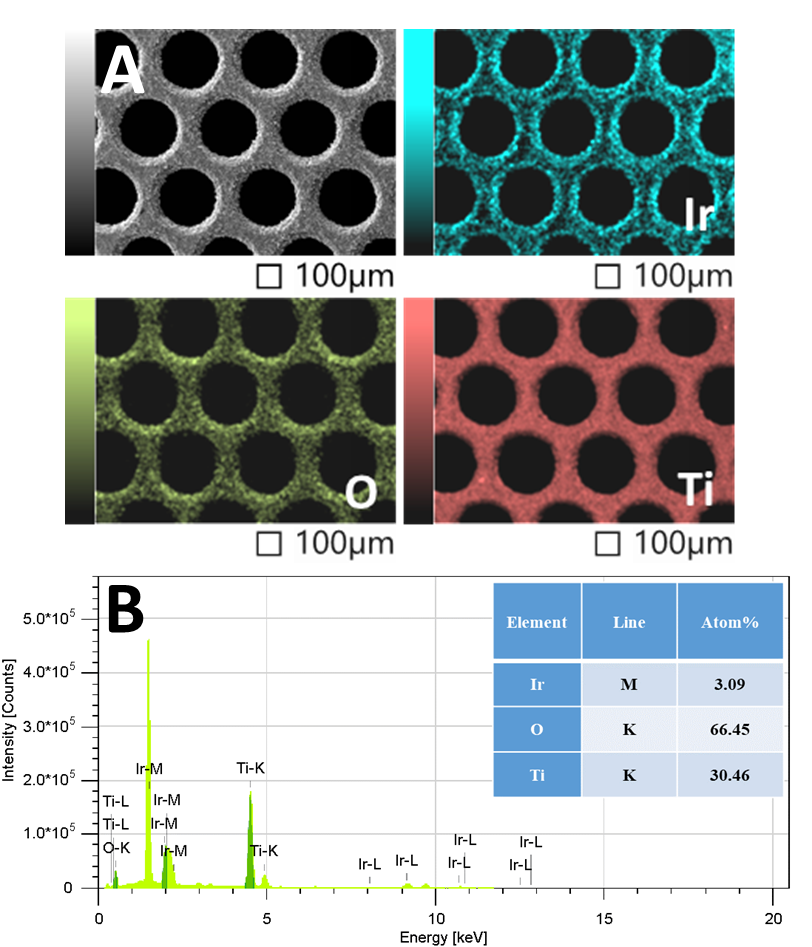
**

**Fig. S2** (**A**) SEM-EDS mapping images of IrO_x_ CCLGDL (0.075 mg cm^-2^); (**B**) EDS analysis of IrO_x_ CCLGDL (0.075 mg cm^-2^)

**
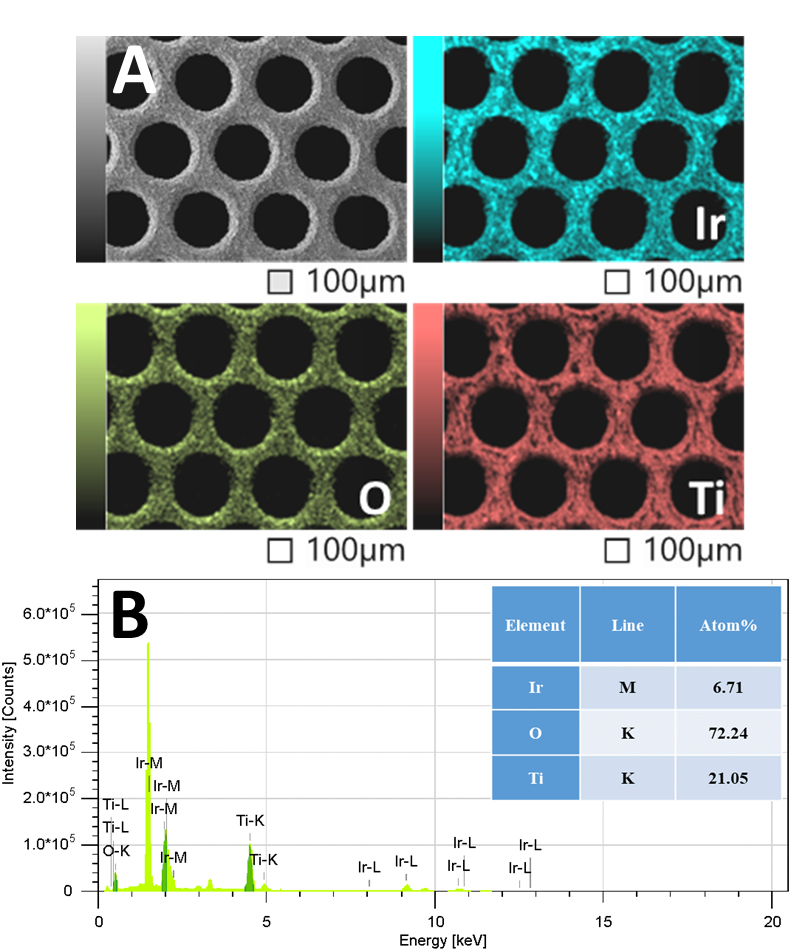
**

**Fig. S3** (**A**) SEM-EDS mapping images of IrO_x_ CCLGDL (0.170 mg cm^-2^); (**B**) EDS analysis of IrO_x_ CCLGDL (0.170 mg cm^-2^)

**
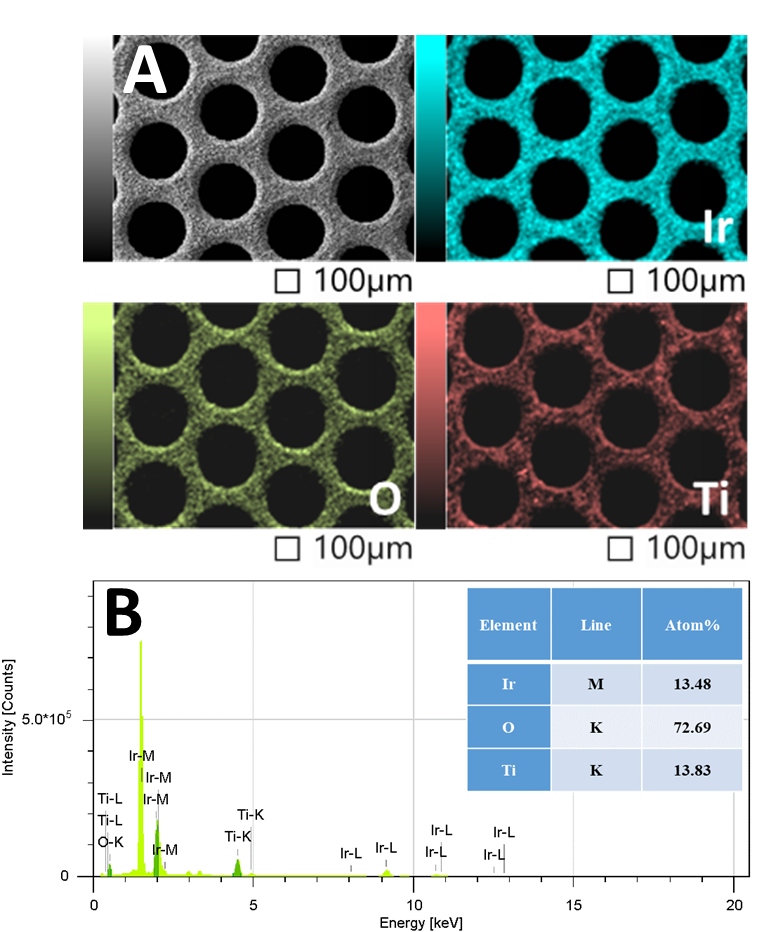
**

**Fig. S4** (**A**) SEM-EDS mapping images of IrO_x_ CCLGDL (0.340 mg cm^-2^); (**B**) EDS analysis of IrO_x_ CCLGDL (0.340 mg cm^-2^)


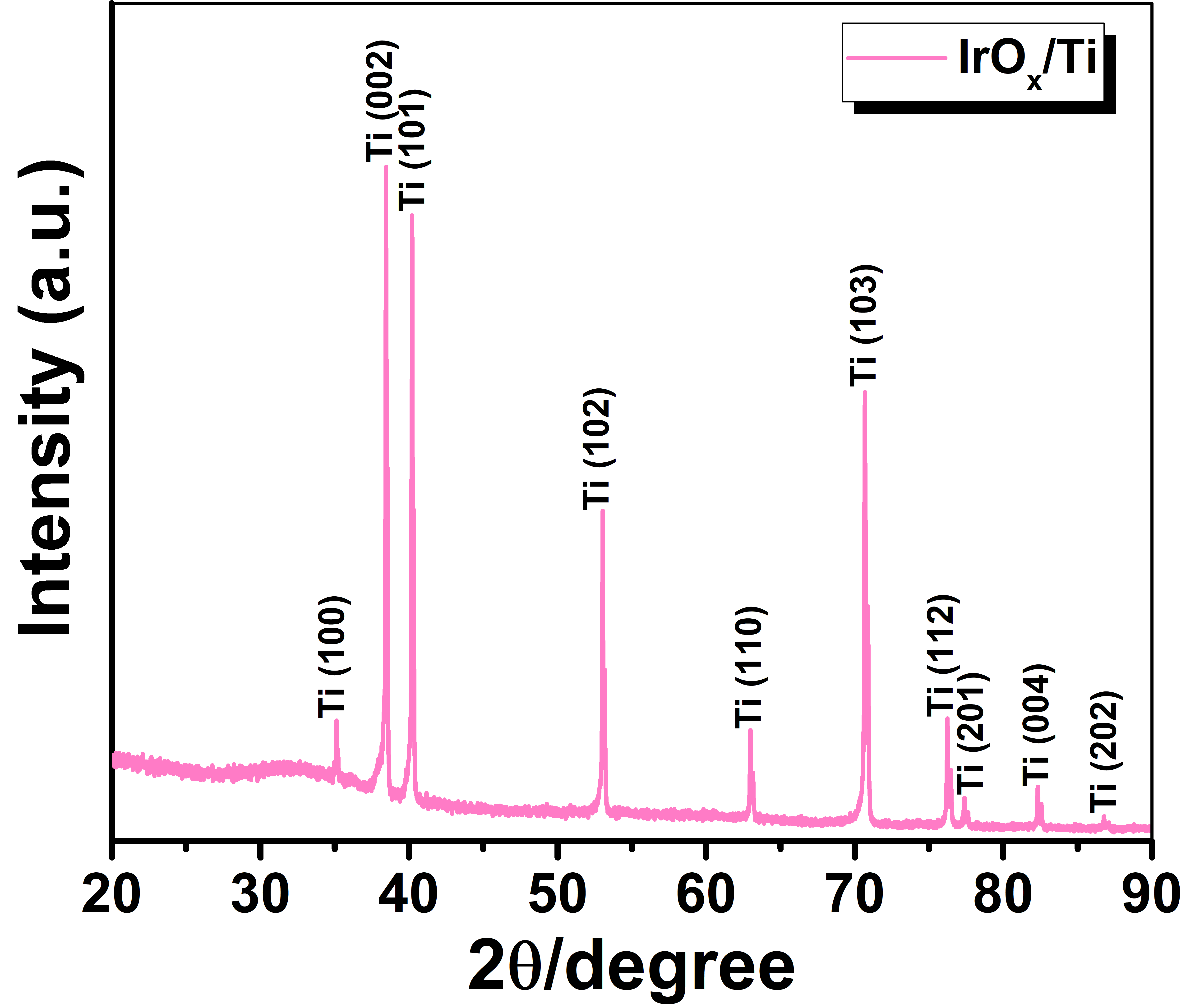


**Fig. S5** XRD pattern of the IrO_x_/Ti


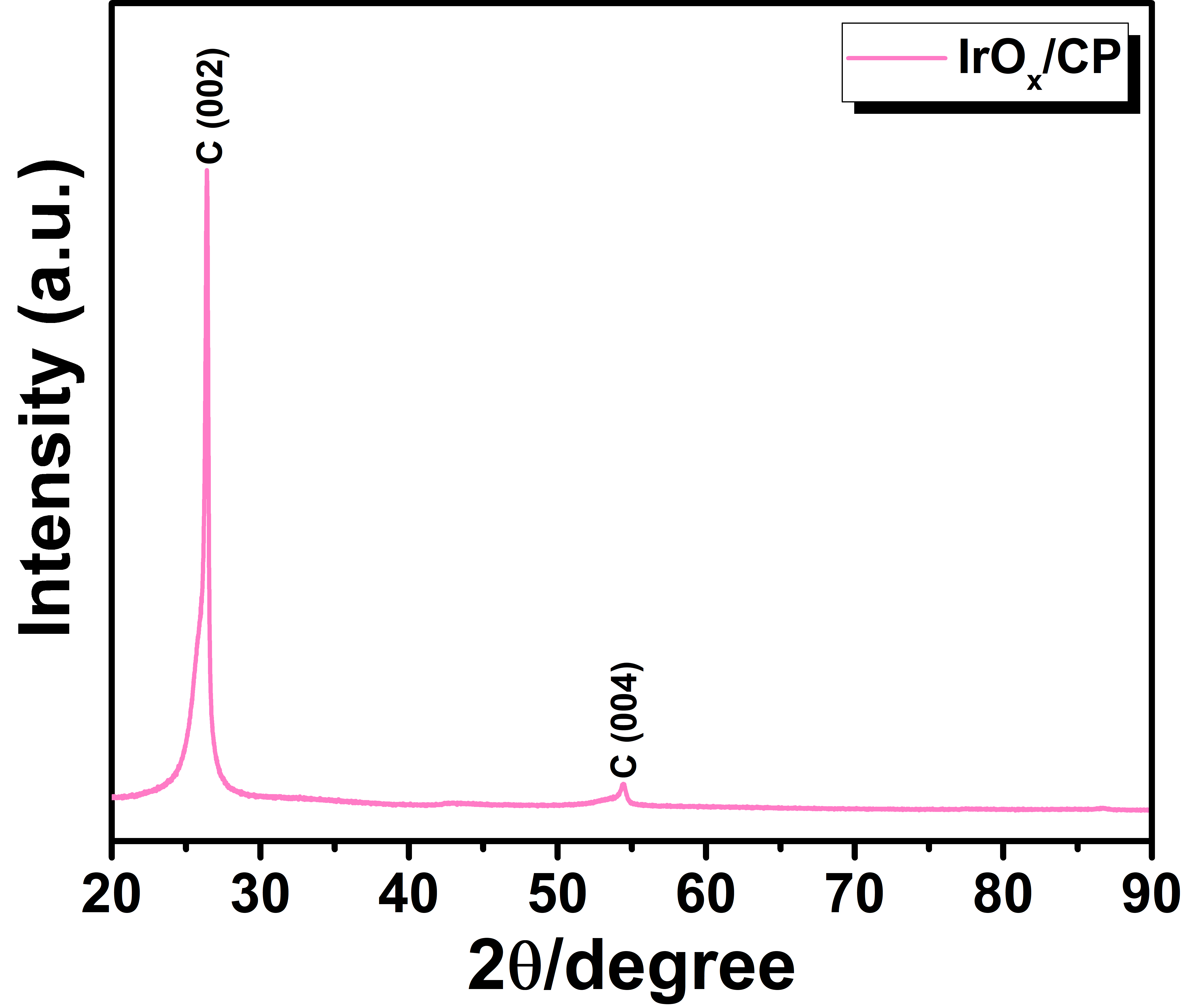


**Fig. S6** XRD pattern of the IrO_x_/CP

**
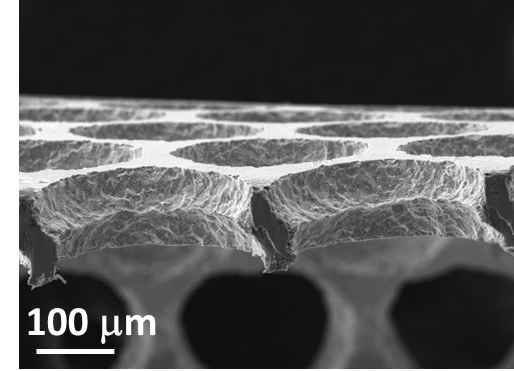
**

**Fig. S7** Cross-section SEM image of the IrO_x_ CCLGDL


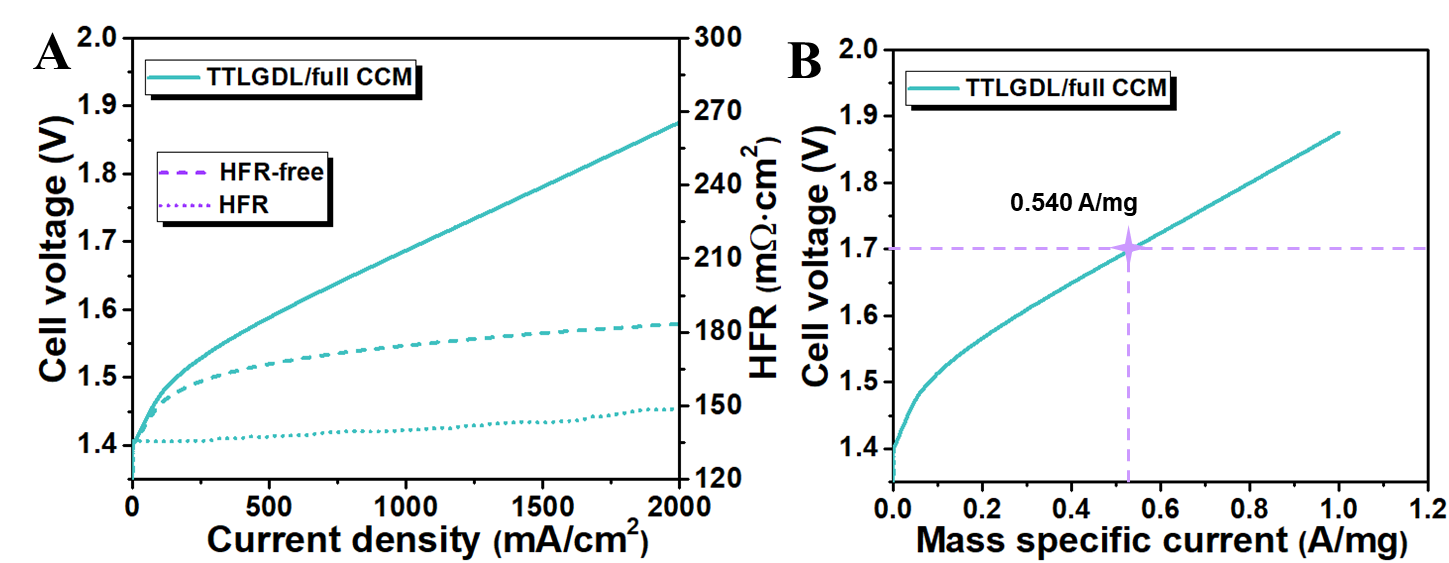


**Fig S8** (**A**) Cell polarization curves and related HFR plot of the TTLGDL coupled with full CCM; (**B**) Ir mass normalized cell polarization curves of the TTLGDL coupled with full CCM

**Table S1** EIS fitting parameters derived from **Fig. 6C**

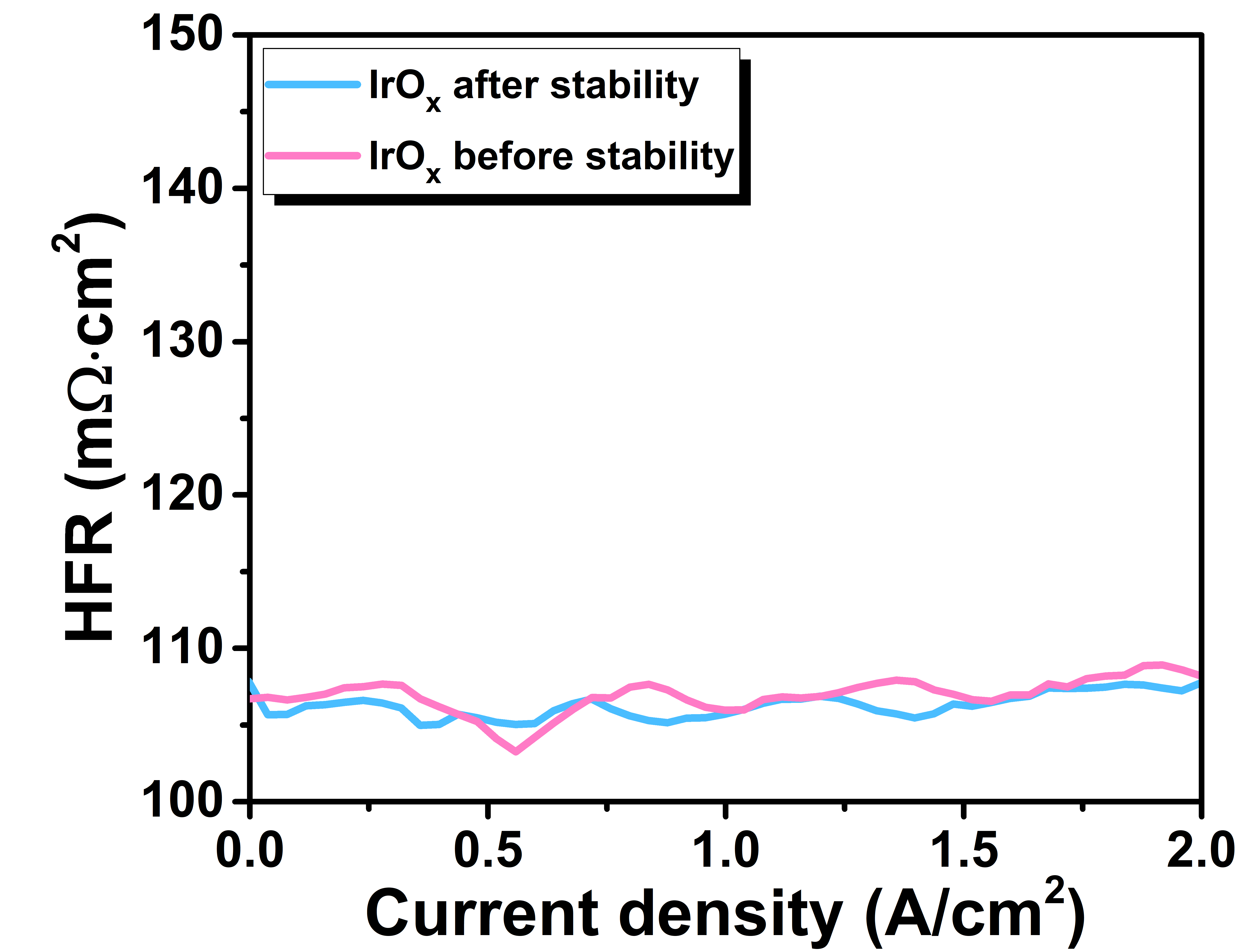


**Fig. S9** HFR plots of IrO_x_ CCLGDL before and after the stability test

**Table S2** Cell test property comparison of previously reported PEMECs with different anodes

| **Catalysts** | **Anode fabrication** | **Catalyst loading (mg cm^-2^)** | **Membrane** | **Voltage (V) at 1 A cm^-2^** | **Voltage (V)**  **at 2 A cm^-2^** | **Mass-specific current at 1.7 V (A mg^-1^)** | **Refs.** |
| --- | --- | --- | --- | --- | --- | --- | --- |
| **0.075-based CCLGDL** | **Electrodeposition** | **0.075** | **N117** | **1.68** | **1.84** | **15.26** | **This work** |
| **0.170-based CCLGDL** | **Electrodeposition** | **0.170** | **N117** | **1.66** | **1.80** | **7.732** | **This work** |
| **0.340-based CCLGDL** | **Electrodeposition** | **0.340** | **N117** | **1.64** | **1.77** | **4.383** | **This work** |
| IrO_2_  (pattern-3) | Commercial/spray | 0.42 | N117 | 1.69 | 1.89 | 2.43 | ACS Appl. Mater. Interfaces 14 (2022) 2335 |
| IrO_2_ | Adams fusion method/  spray | 2.5 | N117 | 1.75 | / | 0.352 | Applied Surface Science 514 (2020) 145943 |
| IrRuO_X_ | Spray-drying+  calcination/  spray + decal | 1.8 | N115 | 1.67 | 1.85 | 0.711 | Adv. Energy Mater. 9 (2019) 1802136 |
| IrO_X_ | Spray-drying+  calcination/  spray + decal | 2 | N115 | 1.68 | 1.85 | 0.575 | Adv. Energy Mater. 9 (2019) 1802136 |
| IrO_2_ nanoneedles | Adams’ fusion method/  spray + decal | 4 | N117 | 1.80 | 2.18 | 0.178 | Adv. Funct. Mater. 2028 (2018) 1704796 |
| IrO_2_ | Hydrothermal reaction/spray | 1.5 | N117 | 1.72 | / | 0.603 | Chem. Eng. J. 419 (2021) 129455 |
| Ir_0.7_Ru_0.3_O_X_  (TT) | Wet chemical method/spray | 1 | N212 | 1.74 | / | 0.835 | Nano Energy 34 (2017) 385 |
| IrO_X_@IrO_2_ (A-450) | Adams’ fusion method/  spray | 1 | N115 | 1.79 | 2.21 | 0.750 | Electrochim. Acta 390 (2021) 138885 |
